# Indirect Evidence for Genetic Differentiation in Vulnerability to Embolism in *Pinus halepensis*

**DOI:** 10.3389/fpls.2016.00768

**Published:** 2016-06-02

**Authors:** Rakefet David-Schwartz, Indira Paudel, Maayan Mizrachi, Sylvain Delzon, Hervé Cochard, Victor Lukyanov, Eric Badel, Gaelle Capdeville, Galina Shklar, Shabtai Cohen

**Affiliations:** ^1^Institute of Plant Sciences, Volcani Center, Agricultural Research OrganizationRishon LeZion, Israel; ^2^Institute of Soil, Water and Environmental Sciences, Volcani Center, Agricultural Research OrganizationRishon LeZion, Israel; ^3^BIOGECO, INRA, Université de BordeauxCestas, France; ^4^PIAF, INRA, Université Clermont AuvergneClermont-Ferrand, France

**Keywords:** embolism, xylem hydraulics, provenance trial, genetic variation, border pit, torus-margo, water potential, xylem conductivity

## Abstract

Climate change is increasing mean temperatures and in the eastern Mediterranean is expected to decrease annual precipitation. The resulting increase in aridity may be too rapid for adaptation of tree species unless their gene pool already possesses variation in drought resistance. Vulnerability to embolism, estimated by the pressure inducing 50% loss of xylem hydraulic conductivity (*P*_50_), is strongly associated with drought stress resistance in trees. Yet, previous studies on various tree species reported low intraspecific genetic variation for this trait, and therefore limited adaptive capacities to increasing aridity. Here we quantified differences in hydraulic efficiency (xylem hydraulic conductance) and safety (resistance to embolism) in four contrasting provenances of *Pinus halepensis* (Aleppo pine) in a provenance trial, which is indirect evidence for genetic differences. Results obtained with three techniques (bench dehydration, centrifugation and X-ray micro-CT) evidenced significant differentiation with similar ranking between provenances. Inter-provenance variation in *P*_50_ correlated with pit anatomical properties (torus overlap and pit aperture size). These results suggest that adaptation of *P. halepensis* to xeric habitats has been accompanied by modifications of bordered pit function driven by variation in pit aperture. This study thus provides evidence that appropriate exploitation of provenance differences will allow continued forestry with *P. halepensis* in future climates of the Eastern Mediterranean.

## Introduction

Climate change, which is leading to increased mean temperatures and, in the Eastern Mediterranean is expected to decrease annual precipitation (Giorgi and Lionello, 2008), may be too rapid to allow adaptation of long lived forest trees, leading to changes in biomes in the near future (Seneviratne, 2012). In order to adapt to climate change, long lived forest tree populations will need genetic variability and/or phenotypic plasticity to survive and reproduce allowing the population to adapt to the new climate conditions. This statement is particularly important for the ability to withstand one to multi-year extreme events, which are already testing our forest species, leading to forest dieback in many regions around the globe (Allen et al., 2010; Choat et al., 2012). It was previously suggested that a rapid climate change requires fast adaptation which relies on existing natural variability rather than on selection of new mutations (Savolainen, 2011). The above considerations have led forestry organizations to consider *in situ* selection of forest trees based on their ability to withstand drought and thrive in environments whose aridity matches that predicted for coming generations (Joyce and Rehfeldt, 2013).

*Pinus halepensis* is widespread in the Mediterranean basin and is one of the most drought-tolerant pine species (Ne’Eman and Trabaud, 2000; Maseyk et al., 2008; Klein et al., 2011, 2014a; Chambel et al., 2013). For that reason, it was selected as the main species for afforestation in semi-arid regions of Israel (Liphschitz and Biger, 2001), which now has the southernmost pine forest in the Mediterranean basin (Schiller, 2000; Rotenberg and Yakir, 2010). Recent increases in tree mortality following two drought periods suggest that the *P. halepensis* plantations are not fully adapted to withstand increasing aridity in the local climate (Dorman et al., 2013).

The fact that *P. halepensis* is spread over various subtropical dry summer to semi-arid climatic zones of the Mediterranean basin suggests that genetic differences exist between local populations (e.g., Schiller et al., 1986; Grivet et al., 2009), and there is considerable interest in finding the best genetic source to use in future plantations. To this end, provenance trials have been carried out at selected sites where seeds from various locations are sown together. These are essential in finding populations harboring desirable traits (White et al., 2007; Chambel et al., 2013). In the trials it is assumed that plant populations that are locally adapted will demonstrate genetic differences in fitness-related traits. Studies on drought resistance through provenance trials have been reported previously for several species including *Pinus* spp. where various parameters have been analyzed in order to determine adaptation to drought stress (Atzmon et al., 2004; Voltas et al., 2008; Eilmann et al., 2013; Gaspar et al., 2013).

Drought resistance is a complex polygenic trait that involves multiple mechanisms at different levels of tissue structure and function, and various tree species utilize different strategies to cope with water shortage (McDowell et al., 2008; Meinzer and McCulloh, 2013; Klein et al., 2014b; Delzon, 2015). Nevertheless, accumulating evidence suggests that drought resistance, in many cases, is well explained by resistance of xylem to embolism (Brodribb et al., 2010; Choat et al., 2012; Barigah et al., 2013; Urli et al., 2013; Delzon and Cochard, 2014). A recent study emphasized the crucial role of embolism resistance in those coniferous species that do not rely on abscisic acid to close stomata (Brodribb et al., 2014).

Conifer xylem consists of overlapping files of elongated narrow tracheids interconnected laterally by bordered pits. The pit and its torus-margo membrane allow efficient water flow, while preventing the spread of emboli by sealing the pit with the torus (i.e., pit aspiration). Early study on bordered pit structure (Sperry and Tyree, 1990) showed the correlation between embolism resistance and bordered pit structure. That study argued that torus flexibility, which was related to the pressure at which pits close (due to torus aspiration into the bordered pit), determines embolism resistance. Later studies suggested that both torus thickness and depth of the pit chamber correlate with greater vulnerability to embolism (Hacke and Jansen, 2009). Recently published articles support the hypothesis that xylem resistance to embolism is a major component of drought resistance in conifers, and suggest that the torus to pit aperture overlap is mechanically related to embolism resistance (Delzon et al., 2010; Bouche et al., 2014).

Due to their importance for drought adaptation, embolism resistance traits are natural candidates in genetic variation studies. It was previously hypothesized that populations from xeric environments would possess greater resistance to embolism than other populations within a species. Using technically advanced methods to measure vulnerability to embolism, it was found that *P. sylvestris* as well as the Mediterranean *P. pinaster* possess low inter-population genetic variation in resistance to embolism (Martínez-Vilalta et al., 2009; Corcuera et al., 2011; Lamy et al., 2011, 2014). A similar study that evaluated Mexican populations of *P. hartwegii* also demonstrated the lack of genetic variability in the embolism resistance trait (Sáenz-Romero et al., 2013). The only pine species that has shown a significant degree of among population genetic variability in embolism resistance so far is *P. canariensis* (López et al., 2013).

A recent study on *P. halepensis* provenances from Israel, Greece, Italy, and Algeria, in three provenance trials reported significant variation in branch hydraulic conductivity and native embolism (Klein et al., 2013). That study supported a previous study that showed higher survival rates of Greek and Israel as compared to Italian and Algerian provenances in semi-arid field trials (Schiller and Atzmon, 2009). In the current study we hypothesized that provenances are genetically different in their hydraulic traits and that these differences are driven by variation in xylem structure. To test these hypotheses, we analyzed hydraulic and anatomical traits of *P. halepensis* in a local provenance trial.

## Materials and Methods

### Plant Material

*Pinus halepensis* provenances used in this study were grown in a provenance trial at Bet Dagan, in the center of Israel, since 1991. Trees sampled were fully grown and 10–15 m tall. Bet Dagan, whose climate is Thermo-Mediterranean, is located in the coastal plain 20 km east of the Mediterranean Sea shore (31°59′N 34°48′E). The site is part of the UN FAO seed collection provenance program (SCM/CRFM/4 bis project^1^). Four provenances were selected for the current research. These included Elea from Greece, Elkosh from Israel, Otricoli from Italy and Senalba from Algeria. Climatic conditions at the native location of the four provenances are indicated in **Table 1** (based on Klein et al., 2013). Mean annual and summer precipitation and approximate potential evapotranspiration at the provenance trial at Bet Dagan were 524, 0, and 1300 mm respectively. Monthly total precipitation and daily pan evaporation covering the sampling period from September 2012 through May 2015 are shown in Supplementary Figure S1.

**Table 1:** Climate data for the four seed source provenances used in this study (based on Klein et al., 2013 and references therein).

Provenance (country)	FAO code	*P*	*P*s	PET	Aridity index
Elea (Greece)	A2	500	60	1350	0.37
Elkosh (Israel)	A7	800	0	1300	0.59
Otricoli (Italy)	A26	830	90	900	0.92
Senalba (Algeria)	A30	310	25	1250	0.25

*P (mean annual precipitation), PET (mean annual potential evapo-transpiration) and Ps (mean summer precipitation) are in mm. Aridity index (P/PET).*

### Specific Hydraulic Conductivity

Light-exposed lower branches containing regular stem sections (i.e., ‘twigs,’ ~20 cm long, 5–8 mm diameter with 5–6 annual rings) were sampled in the morning for hydraulic measurements and several twigs with needle cohorts for measuring leaf water potential (Ψ). Ψ samples were immediately bagged and kept in a cooler during transport to the lab where water potential was measured with a pressure chamber (ARIMAD, MRC Ltd., Holon, Israel). Hydraulic samples were immediately put in an ice bath in the field and remained so during transport to the lab. Resin production was prevented by chilling in the ice bath for between 40 min and 1 h. That led to high conductivity values similar to those obtained by Klein et al. (2013), who boiled sample ends. Twigs were allowed to ‘relax’ for at least an hour before measurement. In the lab more than 2 cm was re-cut from each side of the twig under water and final twig length was about 10 cm. Since pine tracheid length is less than 1 cm this assured that tracheids that cavitated during cutting in the field were not included in the measurements.

Native specific hydraulic conductivity (*K*_s_) and maximum specific conductivity (*K*_smax_) were measured under low pressure (7 KPa) generated by a 70 cm water head before and after overnight perfusion of the xylem tissue with a vacuum at a higher negative pressure of ~0.06 MPa that drew degassed fluid into the samples from a closed container. The vacuum procedure was selected because perfusion at pressures greater than those of the vacuum led to reductions in conductivity, presumably due to pit aspiration. Since a large amount of degassed water was drawn through the stem overnight, we assume that embolisms were refilled, and in fact conductivity after perfusion was much greater. All measurements, including perfusion, were with 0.2 mM KCl solution which was degassed and filtered through Whatman no. 50 (retention of particle size > 2.7 μm) filter paper before use. Hydraulic measurements were made by connecting samples to 25 ml burettes with 0.05 ml resolution and accuracy, allowing measurements with a number of samples in parallel. Readings of the water volume entering the stems from the upstream burette were taken every 20 min to a half hour during which time the water level dropped by less than 4 cm, which we accounted for in the calculations. Water level was readjusted to 70 cm above the water entry point (i.e., the burettes were refilled using a syringe with a long needle) after each reading. Measurement continued for about 2 h until flow rates leveled off. Our protocol has been written up and submitted to the Prometheus Wiki website (*not available yet*). Mean sample stem diameter without bark, and length were measured and specific hydraulic conductivity *K*_s_ and maximum specific conductivity *K*_smax_ (kg m^-1^ MPa^-1^ s^-1^) were calculated after Sperry and Tyree (1988) assuming that all of the stem was conductive. Measurements of *K*_s_ and *K*_smax_ were further used to determine percent loss of conductivity (PLC) that can be attributed to xylem embolism according to Sperry and Tyree (1988). During bench dehydration at very low Ψ (< -6 MPa), in some cases, *K*_smax_ values were low due to insufficient perfusion. In these cases *K*_smax_ measured at the beginning of measurement series when Ψ was higher (> -1.5 MPa) was used.

### PLC Curves

Percent loss of conductivity curves were determined by three methods: bench dehydration (six individual trees within provenance), Cavitron technique (10 individual trees within provenance), and micro-Computed Tomography (micro-CT, five individuals from the Elkosh provenance). With the bench dehydration method (Tyree et al., 1992) using the burette protocol (see above), measurements were made in January, 2014, about a month after a large rainstorm (>100 mm) which saturated the soil. Branches were cut from the trees and these were allowed to dry in the lab or outdoors until they reached the desired needle cohort Ψ. Twigs were then cut from the branches as described above and their specific conductivity was measured. To obtain very low Ψ’s, branches were left outside in the sun for several days. For bench dehydration with the micro-CT, samples were taken in May 2015 and samples were dried on a bench in the lab.

The Cavitron technique (Cochard et al., 2005) was used at the high-throughput phenotyping platform for hydraulic traits (Cavit_Place, INRA-University of Bordeaux, Pessac, France). Branches from 10 individuals per provenance were sent in overnight mail to the above and used for vulnerability curve measurements. *P*_50_ (MPa) was defined as the pressure corresponding to 50% PLC (Lamy et al., 2014). Slope (S), which corresponds to the speed of embolism spread, was defined as the slope (% MPa -1) of a tangent at the inflection point (*P*_50_) as previously described (Lamy et al., 2014).

The X-ray microtomography (micro-CT) technique is a non-invasive observation technique that allows the embolism to be directly visualized (Cochard et al., 2015). Samples were placed in an X-ray microtomograph (Nanotom 180 XS, GE, Wunstorf, Germany) at the PIAF laboratory of the Institut National de la Recherche Agronomique (INRA, Clermont-Ferrand, France) in order to visualize the hydric status of the xylem in different conditions. For one set of measurements, branches similar to the above were measured in the Cavitron and then scanned in the micro-CT system after each centrifugation step. The X-ray settings were adjusted in order to observe the whole cross-section of the middle of the samples with the best spatial resolution. Each scan provided 3D images from which we virtually extracted the central cross-section of the sample with a spatial resolution of 3.75 μm. The rate of embolism was measured by image analysis using ImageJ software^2^. In a second set large branches, including a number of leaf cohorts, were dried by bench dehydration (see above). At different levels of dehydration Ψ was measured in the pressure chamber and branches from the same branch were imaged with the micro-CT.

### Determination of Pit Closing Pressure with a High Pressure Flow Meter (HPFM)

At high water pressures tori of bordered pits are aspirated into the pit borders, thereby sealing the pit aperture and blocking water flow. This behavior has been documented previously for other conifers (Pappenheim, 1889; Sperry and Tyree, 1990). Using a HPFM (Tyree et al., 1995) and small branch sections we found that at low pressures flow reached a steady state and when pressure was increased gradually, at some point the resistance increased steeply, indicating pit closure. Reversing the direction of flow and applying low pressure resulted in a return to the original resistance, indicating that the torus moved out of the pit aperture and the pit opened, which confirms the previous statements. Utilizing the HPFM in this manner, we determined the torus aspiration pressure (or pit closure pressure), i.e., the pressure at which resistance begins to rapidly increase.

Branch sections were connected under water and the HPFM was operated in the steady state mode at a series of increasing low pressures, approximately 10 min per pressure, at intervals of about 0.01 MPa. The pressure at which resistance increased exponentially was taken as the pressure of pit closure (Pappenheim, 1889). In each case 6 samples (from six individual trees) were measured, each 10 cm long and 8–10 mm in diameter. We note that it was difficult to control the HPFM at these low pressures, and we broke a needle valve in the process.

### Tracheid Width Measurement

For anatomical analyses, branch tissue used for hydraulic measurements was fixed in 70% ethanol before sectioning. Sections of 15 μm were prepared with a sliding microtome (Reichert Wien, Shandon, Scientific Company, London) and stained with Safranin O. Sectioned material was viewed under a Leica IM1000 microscope and digital images were taken using a CCD camera (model DC2000, Leica, Germany). Images were later analyzed to determine lumen width of early wood of the preceding year using ImageJ software. A microscopic ruler was used for size calibration. Tracheid width measurements were repeated on independent branches from the same trees used for hydraulic measurements. Two branches per tree and five trees from each provenance were sampled. Approximately 200 tracheids were measured for each tree so that for each provenance about 1000 tracheids were sampled.

### Tracheid Length Measurement

Small segments (toothpick sized) of wood (the outer most second ring) were incubated in maceration solution composed of 1:4:5 of 30% hydrogen peroxide: distilled water: glacial acetic acid, for 3 days and washed five times in distilled water (Peterson et al., 2008). Tracheids were stained with Safranin O and mechanically dispersed before observed and photographed using the microscope. Length was measured using ImageJ software. A microscopic ruler was used for size calibration. Two branches per tree and five trees from each provenance were sampled. Approximately 200 tracheids were measured for each tree so that for each provenance about 1000 tracheids were sampled.

### Scanning Electron Microscopy (SEM)

Branch samples, with 5–6 annual rings, were collected from all provenances and incubated in 70% ethanol. The samples were split in half and small and thin longitudinal sections were cut with a razor blade. These were then oven dried overnight at 65°C. Sections were mounted on aluminum stubs using double sided adhesive and coated with gold-palladium for 90 s at 20 mA using a sputter coater (SC7620 mini sputter coater, Quorum). All samples were observed with a field emission scanning electron microscope (SEM JCM-6000 bench-top scanning electron microscope, JEOL) with an accelerating voltage of 15 kV. Early wood inter-tracheid pit membranes where the pit aperture underneath the torus is clearly visible were photographed. The photos were analyzed to determine torus diameter and pit aperture area using ImageJ software. A minimum 24 pits per provenance were analyzed for torus-aperture overlap [(torus diameter – pit aperture)/torus diameter] following Delzon et al. (2010).

### Statistical Analyses

Results in this study were analyzed using JMP software (SAS Institutes, Inc., Cary, NC, USA). Variations among provenances in water conductivity, PLC curve parameters and xylem anatomical measurements were evaluated using a one-way analysis of variance (ANOVA) followed by Tukey’s Honest Significant Difference (Tukey–Kramer HSD) test. Assessment of phenotypic variability for tracheid width and length was done with a nested ANOVA using the residual maximum likelihood (REML) method. In the nested ANOVA provenances were considered fixed effects and individual trees were nested within provenances as a random effect. Correlations between *P*_50b_ values and anatomical features were tested with the Pearson correlation coefficient (*r*).

## Results

### Differentiation in Hydraulic Traits

Hydraulic conductivity measurements were made in two seasons; at the end of the dry summer season (October 8, 2013) after 170 days with no precipitation and in the middle of the rainy season (January 15, 2014) after rain saturated the soil (Supplementary Figure S1). *Native K*_s_ was lower at the end of the dry season than it was in the rainy season in all provenances (**Table 2**). In both seasons, Elea had the highest native *K*_s_, which was significantly higher than that of the Otricoli and Senalba provenances. Elkosh had intermediate native conductivity which did not differ significantly from the others (**Table 2**). No significant differences in maximum conductivity (*K*_smax_) were found among provenances at the end of the dry season, albeit a tendency for higher conductivity was evident in Elea and Elkosh as compared to Otricoli and Senalba (**Table 2**). Similar results of *K*_smax_ were obtained with the Cavitron at the beginning of the rainy season of 2014 (with less than 20 mm precipitation, Supplementary Figure S1), after 145 days with no precipitation. A significant difference was found in the rainy season between the high *K*_s__max_ of Elea and the low *K*_s__max_ of Otricoli (**Table 2**).

**Table 2:** Mean values (±SE) of specific conductivity (Ks, kg m^-1^ MPa^-1^ s^-1^) of stems and tracheid dimensions of the four *P. halepensis* provenances.

	End of dry season, 2013		Middle of rainy season, 2014				End of dry season – 2014
Provenance	*K*_s_ native	*K*_s_ max	*K*_s_ native	*K*_s_ max	Tracheid width, μm	Tracheid length, μm	K_s_ max
Elea	0.20 ± 0.03^a^	0.24 ± 0.04	0.28 ± 0.04^a^	0.29 ± 0.04^a^	18.2 ± 0.1	1821 ± 16.7	0.56 ± 0.05
Elkosh	0.16 ± 0.01^ab^	0.22 ± 0.02	0.21 ± 0.02^ab^	0.22 ± 0.02^ab^	17.1 ± 0.1	1715 ± 16.7	0.58 ± 0.05
Otricoli	0.12 ± 0.01^b^	0.19 ± 0.02	0.15 ± 0.01^b^	0.16 ± 0.01^b^	17.0 ± 0.1	1761 ± 16.7	0.52 ± 0.06
Senalba	0.10 ± 0.01^b^	0.17 ± 0.01	0.16 ± 0.01^b^	0.19 ± 0.02^ab^	17.4 ± 0.1	1674 ± 16.9	0.49 ± 0.07
*P-value*	<0.003	<0.16	<0.003	<0.013	<0.6	<0.5	<0.7

*Measurements were made at the end of the dry season (2013) and in the middle of the winter rainy season (2014) using the bench dehydration method (n = 5), and at the end of the dry summer season of 2014 with the Cavitron technique (n = 10). Significant differences between means are indicated by different lowercase letters.*

**FIGURE 1:**
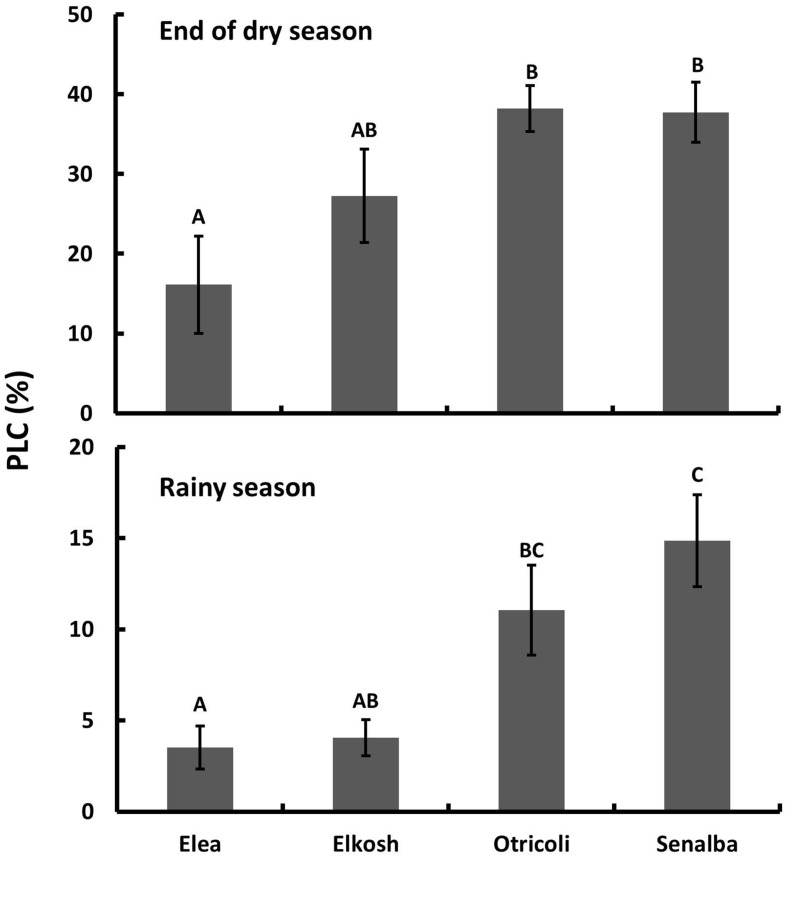
**Percent loss of conductivity (PLC) of *P. halepensis* provenances measured at the end of the dry season (upper) and in the middle of the rainy season (lower).** Significant differences between provenances are indicated by different letters (*n* = 5, *P* < 0.05).

Elea and Elkosh provenances had low percent loss of conductivity in both seasons (**Figure 1**). Elea had significantly lower PLC (16.1 ± 6.1 and 3.5 ± 1.2 % at the end of the dry and in the rainy seasons, respectively) than Senalba (37.7 ± 3.8 and 14.9 ± 2.5%) and Otricoli (38.2 ± 2.9 and 11.1 ± 2.5%). Elkosh had low PLC (27.2 ± 5.8 and 4.1 ± 1.0%), which was similar to Elea, but its PLC was not statistically different from Otricoli and Senalba in the dry season and from Otricoli in the rainy season (**Figure 1**).

Vulnerability curves were measured with the bench dehydration and Cavitron methods (Supplementary Figure S2). All methods and provenances showed similar shapes of vulnerability curves and data points were fit to a sigmoidal model (Pammenter and Van der Willigen, 1998). Notable in the curve fit for bench drying (Supplementary Figure S2, upper panel) is that for the lowest water potentials, -8 MPa, some conductivity remained, and extrapolated values for 100% loss of conductivity are very low, close to -10 MPa, which was the lower limit of the pressure chamber used for Ψ measurements. For bench drying, Senalba and Otricoli provenances had the highest *P*_50_, -3.6 ± 0.04 and -3.7 ± 0.1 MPa, respectively, and Elkosh and Elea were lower, -4.2 ± 0.1 and -4.5 ± 0.1, respectively, indicating higher embolism resistance in Elkosh and Elea (**Table 3**).

**Table 3:** Mean values (±SE) of embolism resistance parameters of the four *P. halepensis* provenances as measured by the Cavitron (subscript c) and bench (subscript b) drying methods.

provenance	*P*_c,12_	*P*_b,12_	*P*_c,50_	*P*_b,50_	*P*_c_*_,_*_88_	*P*_b_*_,_*_88_	Slope_c_	Slope_b_	Torus-aperture overlap
Senalba	-4.21 (±0.26)	-1.2 (±0.06)^a^	-5.04 (±0.24)^a^	-3.6 (±0.04)^a^	-5.9 (±0.3)^a^	-6.1 (±0.2)^a^	61 (±11)^a^	20.3 (±0.4)^a^	2.31 (±0.06)^a^
Otricoli	-4.25 (±0.29)	-0.97 (±0.07)^a^	-5.08 (±0.28)^a^	-3.7 (±0.1)^a^	-5.9 (±0.4)^a^	-6.4 (±0.07)^a^	63 (±12)^a^	18.6 (±0.4)^b^	2.50 (±0.08)^a^
Elea	-4.51 (±0.32)	-1.8 (±0.04)^c^	-5.27 (±0.34)^ab^	-4.5 (±0.08)^c^	-6.0 (±0.4)^a^	-7.2 (±0.06)^c^	68 (±16)^a^	18.6 (±0.3)^b^	3.31 (±0.16)^b^
Elkosh	-4.36 (±0.33)	-1.5 (±0.06)^b^	-5.51 (±0.39)^b^	-4.2 (±0.09)^b^	-6.7 (±0.6)^b^	-6.8 (±0.1)^b^	46 (±010)^b^	18.7 (±0.3)^b^	3.05 (±0.12)^b^
*P-value*	<0.2	<0.001	<0.02	<0.001	<0.02	<0.001	<0.002	<0.005	<0.004

*P_12_, P_50_, and P_88_ indicate pressures causing 12, 50, and 88 percent loss of conductivity. Slope is at the inflection point of the vulnerability curve. Significant differences between means in each column are indicated by different lowercase letters.*

Vulnerability curves measured by the Cavitron technique suggested a similar tendency of variation with more negative values. *P*_50_ of Elkosh (-5.51 ± 0.39) was significantly lower than that of Senalba (-5.04 ± 0.24) and Otricoli (-5.08 ± 0.28) but not of Elea (-5.27 ± 0.34). *P*_50_ of Elea was not significantly lower than that of Senalba or Otricoli (**Table 3**). Substantially lower *P*_88_ was observed in Elkosh (-6.7 ± 0.6) as compared to the other three provenances, while differences in *P*_12_ were small and not significant. Consequently, the slope of the vulnerability curves was significantly lower for Elkosh than for the other provenances (Supplementary Figure S2; **Table 3**).

Differences between the curves measured with the bench drying and Cavitron methods were large and significant. On average *P*_12_, *P*_50_, and *P*_88_ values were 3.0, 1.2, and 0.5 MPa higher, respectively, for the bench drying method, and slopes for the Cavitron were 40%/MPa higher. Thus the largest discrepancy between the methods is in their estimate of the onset of the loss of conductivity at high xylem pressure.

**FIGURE 2:**
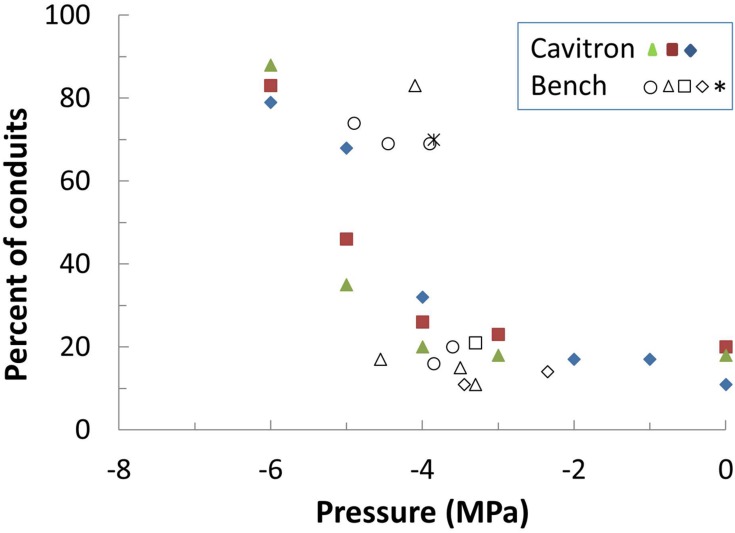
**Percent of empty xylem conduits counted on the micro-CT images of Elkosh samples brought to different pressures by the Cavitron (full symbols) and bench dehydration (empty and star symbols).** Cavitron pressures are calculated from rotor speed while pressures for bench dehydration are measured with a pressure chamber. Each point represents one image. Five branches were measured in the dehydration set and three in the Cavitron. Each branch is plotted with a different symbol.

**FIGURE 3:**
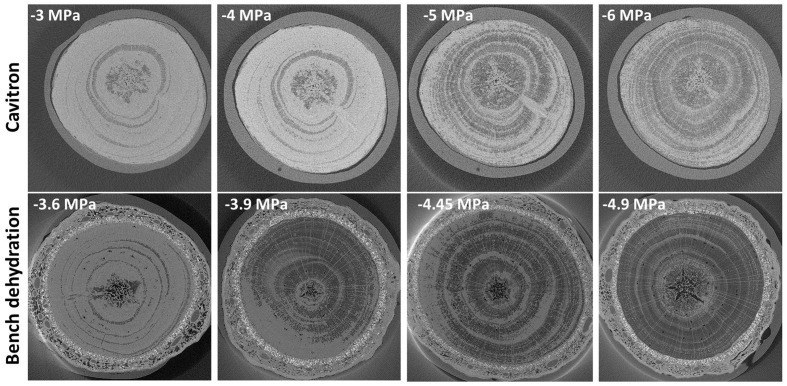
**Micro-CT images of Elkosh provenance at different tensions as induced by centrifugation with the Cavitron (upper row) and by the bench dehydration method (lower row).** Each horizontal panel represents one tree for which segments of 28 cm long with 1 cm diameter were scanned at the middle of the sample.

Bench drying and the centrifuge technique were each used to bring branches from the Elkosh provenance to a given xylem pressure and then they were directly visualized via micro-CT technology (**Figure 3**; Supplementary Figure S3). Empty tracheids do not absorb x-rays and appear as dark spots on x-ray images, while water in the fully saturated tracheids appears gray. Thus, an image segmentation allowed to distinguish the embolized area from the conductive areas and to compute the rate of embolism based on hydraulic calculations using tracheid dimensions (Cochard et al., 2015). Results show that from 0 to -3.6 MPa only 10–20% of the conduits were empty and remained very close to the native embolism. For bench drying most conduits cavitated abruptly at about -3.9 MPa. For the centrifuge technique, embolism was more gradual, began at about -4 MPa and reached *P*_50_ at a value around -5 MPa (**Figures 2** and **3**). The results for *P*_50_ (from conductivity) are in agreement with the other bench drying and Cavitron sets, which gave values of -4.2 and -5.5 MPa, respectively (**Table 3**). The slopes from the micro-CT set as well as the lack of change in embolism from 0 to -4 MPa are closer to the results obtained with the Cavitron technique.

**FIGURE 4:**
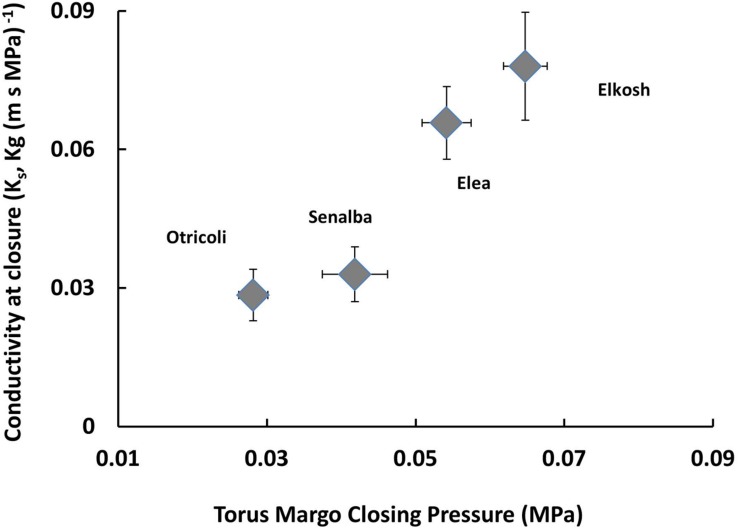
**Pressure and specific conductivity at the point of the beginning of Torus-margo (valve) closure as measured by the HPFM**.

**Figure 4** shows pit closure pressures plotted against the conductivity obtained before pit closure. The results show that Elkosh and Elea had higher closure pressure as compared to Senalba and Otricoli, which may also indicate of an adaptation to aridity (Sperry and Tyree, 1990).

### Differentiation in Xylem Anatomy

No differences in both lumen width and tracheid length were found among provenances when within-population variation was taken into account. However, Elea had the widest tracheids (18.2 ± 0.1 μm; **Table 2**), and tracheids of Elea (1.82 ± 0.02 mm) were longer than those of Elkosh (1.71 ± 0.02 mm) and Senalba (1.67 ± 0.01 mm) but not of Otricoli (1.76 ± 0.02 mm, **Table 2**).

**FIGURE 5:**
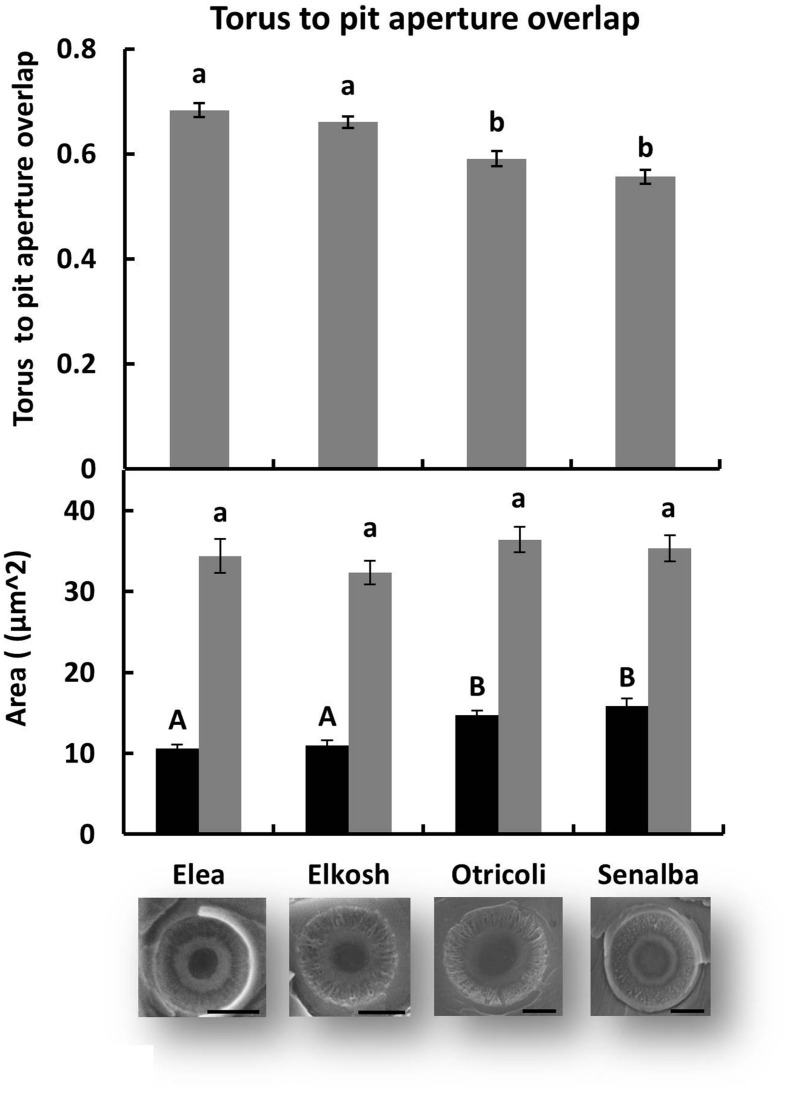
**Torus and pit aperture sizes**. Torus to pit aperture overlap **(upper histogram)**, and area of torus versus pit aperture **(lower histogram)**. Significant differences between provenances are indicated by different letters (*p* < 0.05). Examples of electron microscope images of pits are shown below. (bars = 5 μm).

Measurements of torus-aperture overlap, shown in **Figure 5**, indicate that Elea and Elkosh had similar torus-aperture overlaps (0.68 ± 0.01 and 0.66 ± 0.01, respectively), which were significantly higher than those of Otricoli and Senalba (0.59 ± 0.01 and 0.56 ± 0.01, respectively). These differences were due to smaller pit apertures in Elea and Elkosh, while torus area was similar in all provenances (**Figure 5**). Torus-aperture overlap results were in agreement with pit closure pressures.

Pearson’s correlation coefficients for the relationships between *P*_50b_ values and anatomical features for the four ecotypes were significant for pit aperture area and torus overlap (*p* < 0.05), but not for torus area.

## Discussion

This study evaluated differences and relationships between xylem hydraulic traits and anatomy in four *P. halepensis* provenances in a provenance trial. We found differences in both native and maximum (saturated) xylem hydraulic conductivity that were season-dependent and did not correlate with tracheid dimension. A significant correlation was found between resistance to embolism and bordered pit structure.

### Differences in Hydraulic Traits and Tracheid Size

Klein et al. (2013) reported differences in native PLC in the provenances studied here. Our results are in general agreement with theirs, but we found different PLC values and lower variation between measurements (i.e., better repeatability), probably due to improved technique.

Native *K*_s_, as opposed to *K*_smax_, reflects the actual hydraulic conductivity of the tree in the field, which is probably influenced by cumulative embolism caused by climate events which reduce Ψ’s below the threshold for embolism, and depends on the age of the branch (Bucci et al., 2007; Choat et al., 2012). The significant differences between the high *K*_s_ of Elea and the low *K*_s_ of Senalba and Otricoli provenances, at the end of the dry season, probably result from losses of conductivity due to embolism, since differences in *K*_smax_ were not significant and all samples were of approximately the same age (**Table 2**). Although not significant, the subtle differences in *K*_smax_ favoring Elea and Elkosh over Otricoli and Senalba were consistent through all measurements by both methods. It has been previously suggested that tracheid dimensions explain most of hydraulic conductivity variation in conifers (Hacke and Jansen, 2009). The relationship between tracheid width and xylem conductivity is easy to estimate if we assume that tracheid density was constant in the branches. Then, according to the Hagen-Poiseuille relationship, differences in conductivity are proportional to the fourth power of tracheid diameter (Tyree and Zimmermann, 2002; Pickard and Melcher, 2005). For the two extremes, Otricoli and Elea, the increase in tracheid diameter from 17 to 18.2 μm should increase conductivity by 24%, while the measured *K*_smax_ was 0.19–0.24 kg m^-1^ MPa^-1^ s^-1^, respectively, a change of 26% (**Table 2**), indicating good agreement. However, with the four provenances we have studied, we did not find correlation between *K*_smax_ and tracheid width. Nonetheless, in order to determine correlation between *K*_smax_ and tracheid width, a broad survey of many more provenances is necessary. Significant differences in *K*_smax_ in the middle of the rainy season, but not at the end of the dry season (**Table 2**), may indicate differences in the timing of cambial activity, which may be earlier in Elea than in Otricoli. Indeed, trunk growth initiation is earlier in Elea than in Otricoli (Klein et al., 2013). Differences in phenological events in a provenance trial may indicate genetic differences.

Elea and Elkosh, which had slightly higher hydraulic conductivity, had lower native PLC than Otricoli and Senalba in both seasons (**Figure 1**). Thus, xylem safety does not seem to depend on hydraulic conductivity. These results are in agreement with many other studies that have shown weak or no correlation between conducting efficiency and embolism resistance in conifers (Pockman and Sperry, 2000; López et al., 2002, 2013; Choat et al., 2007; Bouche et al., 2014; Guillermina et al., 2014).

### PLC Curves Measured with the Two Methods

The PLC curve results obtained here by the bench dehydration method (Supplementary Figure S2) gave *P*_50_ values between -3.6 and -4.5 MPa for the four provenances, which is lower than the value reported for *P. halepensis* by Oliveras et al. (2003) using the air injection method, -3.1 MPa, but substantially higher than the values obtained with the Cavitron (Supplementary Figure S2), i.e., -5.9 to -6.7 MPa, that were similar to previous Cavitron *P. halepensis* values (Delzon et al., 2010). Curves relating *P*_50_ values to minimum Ψ (which define safety margins) can also be used to estimate an expected *P*_50_ value (Meinzer et al., 2009). Based on the observations of minimum Ψ’s of between -3 and -4 MPa in the summer in the arid Yatir forest (Klein et al., 2011), and using a relationship based on data from other conifers, the expected value of *P*_50_ is less than -6 MPa (Meinzer et al., 2009), which lends support to the Cavitron measurements. However, it might be argued that the values analyzed by Meinzer et al. (2009) were measured with centrifuge-based methods, similar to the Cavitron technique.

The discrepancy between centrifuge based measurements, as represented here by the Cavitron technique, and the bench dehydration and air injection methods is too large to smooth over. As noted recently, a number of conflicting results from different methodologies used in plant hydraulics need attention (Jansen et al., 2015). Our case is one of them, and putting the measurement methods together, i.e., bench dehydration, Cavitron technique and validation with micro-CT can add some important insight. One surprise is that the micro-CT observations confirmed that the *P*_50_ determinations for both methods of cavitating branches do, in fact, correspond to approximately 50% embolism, even though the values of Ψ differed, on average, by 1.2 MPa. One possible explanation is that the water potential determined in the two methods is not equivalent, i.e., that obtained from rotor speed is not the same as that obtained in the pressure chamber. It is important to note that for centrifuge method the pressures in the branch are not equal at different positions along the branch. Equivalence of centrifuge and pressure chamber measurements has been demonstrated before for broadleaved stems (Holbrook et al., 1995), but perhaps some peculiarity of *P. halepensis*, e.g., it’s very short tracheids, causes differences. Other explanations are possible, and our results call for more experimentation and analysis. As demonstrated here, the Micro-CT method provides an opportunity for direct validation of some of the results and we expect that further exploitation of this method will bring us closer to the ‘truth.’

One important implication of the different results is with respect to evidence for seasonal differences in PLC, as found here (**Figure 1**). Actual soil water potential at which stomata close is reflected by summer needle Ψ, which has been shown to range from -2.4 to -3.7 MPa in *P. halepensis*, close to other measurements of leaf Ψ in summer (Schiller and Cohen, 1998; Klein et al., 2011), and those values should be close to the minimum Ψ in branches. *P*_b,12_ values (**Table 3**) from bench drying measured by the pressure chamber suggest that embolism can be well above 12% even when Ψ is above -4 MPa (**Table 3**), in agreement with the PLC values in **Figure 1**. On the other hand, if the onset of embolism is significantly lower than -4 MPa, as indicated by the *P*_c,12_ values (**Table 3**), it is hard to explain why PLC was much higher in the summer in our conditions (**Figure 1**).

Significant differences in the slope of PLC curves were found in the provenances, and both methods found that Elkosh was significantly lower than Senalba (Supplementary Figure S2; **Table 3**). Similar results were reported for *P. canariensis*, *P*_12_ being similar in all populations studied, whereas *P*_50_, *P*_88_ and slope showed statistically significant differences (López et al., 2013). A less steep slope suggests that embolism occurs gradually over a larger Ψ range, resulting not only in less vulnerability but in a greater safety margin between stomatal closure and catastrophic embolism.

### The Correlation of Pit Aperture Area with Embolism Resistance

*P*_50_ values of Elea and Elkosh indicate that they are more resistant to embolism than Otricoli and Senalba (**Table 3**). The variations in *P*_50_ correlated with variations in torus to pit ratio that were associated with variations in pit aperture area and not with torus area (**Figure 5**). The dominant effect of the pit aperture size rather than the torus membrane size was also demonstrated in two broad surveys of coniferous species, which suggested that embolism resistance parameters are strongly correlated with pit apertures, whereas only a weak correlation was found with torus diameter (Delzon et al., 2010; Bouche et al., 2014). Interestingly, modifications in pit aperture have been observed in tracheids along the 85 m trunk of Douglas-fir where pit aperture decreases with height, whereas torus diameter remains relatively constant (Domec et al., 2008). That modification along the trunk demonstrates a tradeoff between xylem safety and water conducting efficiency ensuring maximum height in Douglas-fir trees (Domec et al., 2008). Variation in pit aperture size and not in torus size suggests that independent developmental mechanisms control the size of these two elements and that genetic differentiation in border pit function are driven by variation in pit aperture size. Differences in pit closure pressures (**Figure 4**), which showed that the more resistant provenances closed at higher pressure, are an additional indication of functional anatomical adaptations.

The results of the current study are supported by the high survival rates of Elea and Elkosh in comparison to Otricoli and Senalba provenances that failed to survive in a more arid provenance trial in Yatir forest (Atzmon et al., 2004; Schiller and Atzmon, 2009). It seems that Elea and Elkosh are better adapted to drought conditions as they allow better growth performance, and also ensure embolism resistance by high torus to pit overlap. All together, these results imply the possibility to predict provenance performance under drought conditions from their structure and performance under more optimal conditions.

### Genetic Differences in Resistance to Embolism

In the current study we have tested only four provenances of *P. halepensis* that were divided into two groups by means of embolism resistance. It has been demonstrated that in general as well as for gymnosperms specifically, genetic diversity is higher in the eastern than in the western Mediterranean (Fady and Conord, 2010). Using chloroplast simple sequence repeats (SSR) markers, Grivet et al. (2009) demonstrated higher genetic diversity in eastern populations of *P. halepensis* in comparison to more western ones. Particularly, it has been shown that Elea, the Greek population, is genetically different from the other populations in that study, and that Shaharia, the Israeli population (represented here by the Elkosh provenance) is significantly different from those of Algeria and Morocco (Grivet et al., 2009). The alignment of the two eastern provenances Elkosh and Elea as being more embolism resistant might relate to their high genetic diversity in comparison to Otricoli and Senalba provenances, which represent the Western populations and demonstrate low embolism resistance. Still, in order to test the relationship between the embolism resistance traits on the genetic diversity level, it is necessary to analyze more populations of *P. halepensis* from various regions for their ability to resist embolism and for their level of genetic variation.

The other *Pinus* species that had been shown to possess intraspecific variation of embolism resistance so far is *P. canariensis* (López et al., 2013). Both *P. halepensis* and *P. canariensis* are the southernmost pine species of the northern hemisphere, colonizing a wide range of climates, and are thus considered the most drought-resistance pines. It appears that both species possess populations that experience extreme suboptimal climatic conditions.

In contrast to *P. halepensis* and *P. canariensis*, no or limited intraspecific variation in embolism resistance was evident in *P. sylvestris* (Martínez-Vilalta et al., 2009). The wide distribution of *P. sylvestris* mostly comprises boreal regions, but it also includes dry areas such as sites in Turkey. However, the reported limited genetic variation in embolism resistance did not include xeric sites in that study (Martínez-Vilalta et al., 2009). Limited intraspecific variation in embolism resistance was also reported in *P. pinaster* (Corcuera et al., 2011; Lamy et al., 2011, 2014). Similar to *P. halepensis*, *P. pinaster* is considered a Mediterranean pine, although its distribution is restricted to the western part of the Mediterranean Basin and its habitat also includes the Atlantic coast (Bucci et al., 2007). It was suggested that the two species represent contrasting biogeographic and demographic histories that probably had strong effects on variation in drought resistant traits (Gómez et al., 2005). Lamy et al. (2014) suggested that embolism resistance in *P. pinaster* is a canalized trait, meaning that the trait stays stable under various environmental conditions. The ability of an organism to canalize a trait depends on internal genetic factors, and that ability is developed by natural selection (Waddington, 1942). Therefore, it might be that different populations would express greater or lesser extent of canalization to a certain trait, depending on time of separation and on various genetic and environmental factors. The significant but conservative difference in *P*_50_ (less than 1 MPa) between populations in the current study might imply a degree of canalization strength in the embolism resistance trait. Similar moderate differences in *P*_50_ were also demonstrated in *P. canariensis* (López et al., 2013). It is probable that natural developmental variation in pit aperture was triggered by extreme climatic events that are more present in semi-arid areas with a long dry season, as is the situation in some habitats of *P. halepensis* and *P. canariensis* (López et al., 2013; Dorman et al., 2015).

## Conclusion

Our results showed significant differentiation in embolism resistance among *P. halepensis* in a provenance trial. This observation was consistently found using three different methods, i.e., bench drying, Cavitron technique and micro-CT. These differences were supported by anatomic analysis suggesting that pit aperture size is a key feature in determining embolism resistance. Although moderate, the observed natural variation in the embolism resistance trait in *P. halepensis* might be sufficient to promote adaptation to climate change. In the light of our results we speculate that species that are subjected to a wide range of climates, including extreme dry environment would express a lesser extent of canalization in embolism resistance traits than species that grow in a more moderate climate range. We therefore suggest including populations that grow at sites with sub-optimal climate conditions in future studies in order to detect genetic variation in the embolism resistance trait.

## Author Contributions

RD-S and SC designed the research. RD-S analyzed the anatomical measurements and wrote the manuscript. IP and VL performed the bench dehydration hydraulic measurements and IP analyzed the data. MM and GS performed the anatomical measurements. SD and GC performed and analyzed the Cavitron measurements. HC and EB performed and analyzed the micro-CT measurements. RD-S, SC, SD and HC revised the manuscript. All authors carefully read and approved the final manuscript.

## Conflict of Interest Statement

The authors declare that the research was conducted in the absence of any commercial or financial relationships that could be construed as a potential conflict of interest.

## Footnotes

1 http://www.fao.org/docrep/006/k1203e/K1203E08.htm

2 http://rsb.info.nih.gov/ij/
