# Supplementary figures and images for: Indirect Evidence for Genetic Differentiation in Vulnerability to Embolism in Pinus halepensis
Source: Front Plant Sci. 2016 Jun 2;7:768. doi: 10.3389/fpls.2016.00768 (PMC4889591; doi:10.3389/fpls.2016.00768)

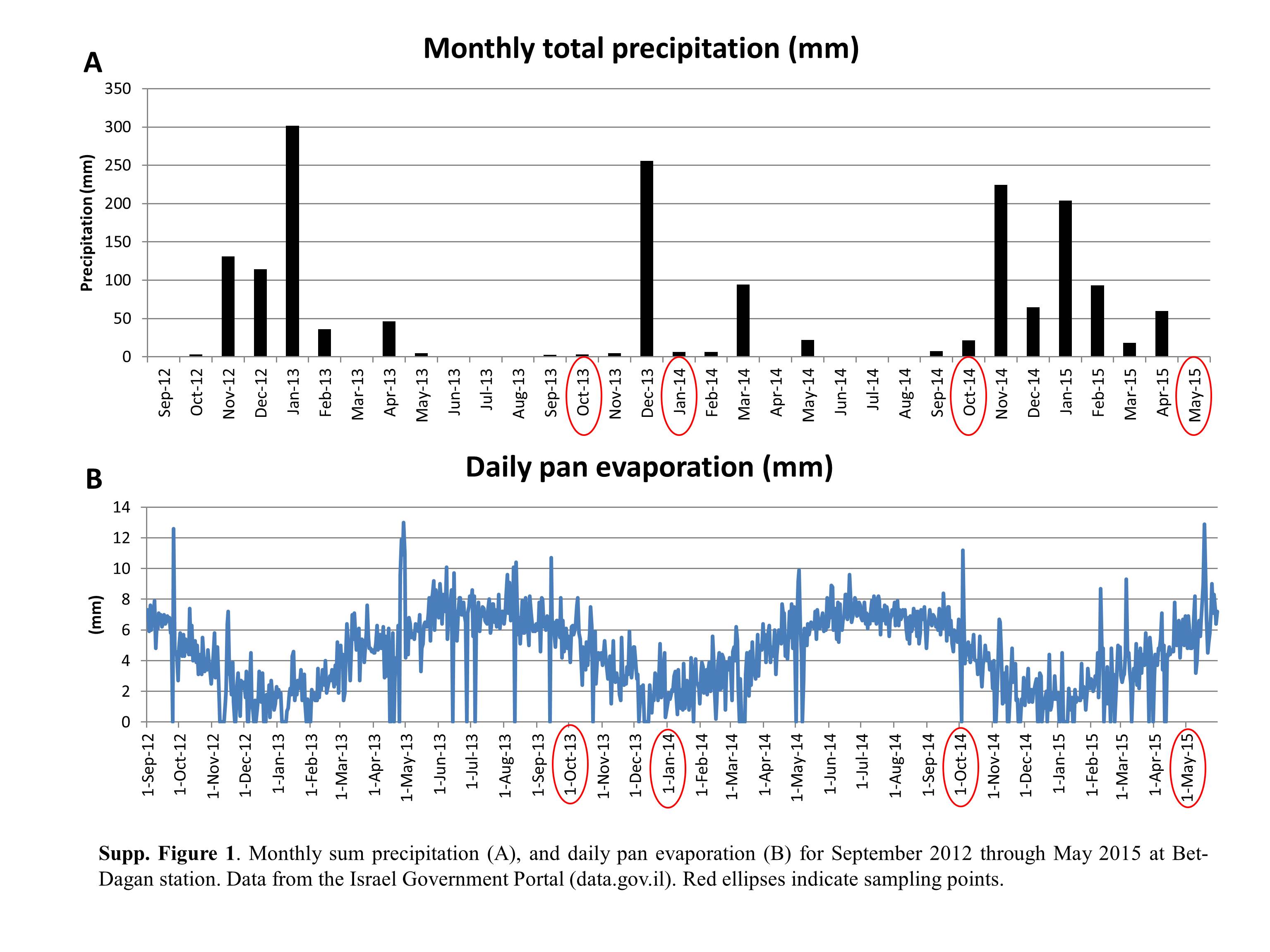

Supplement: Supplementary file 1 [file Image_1.JPEG]

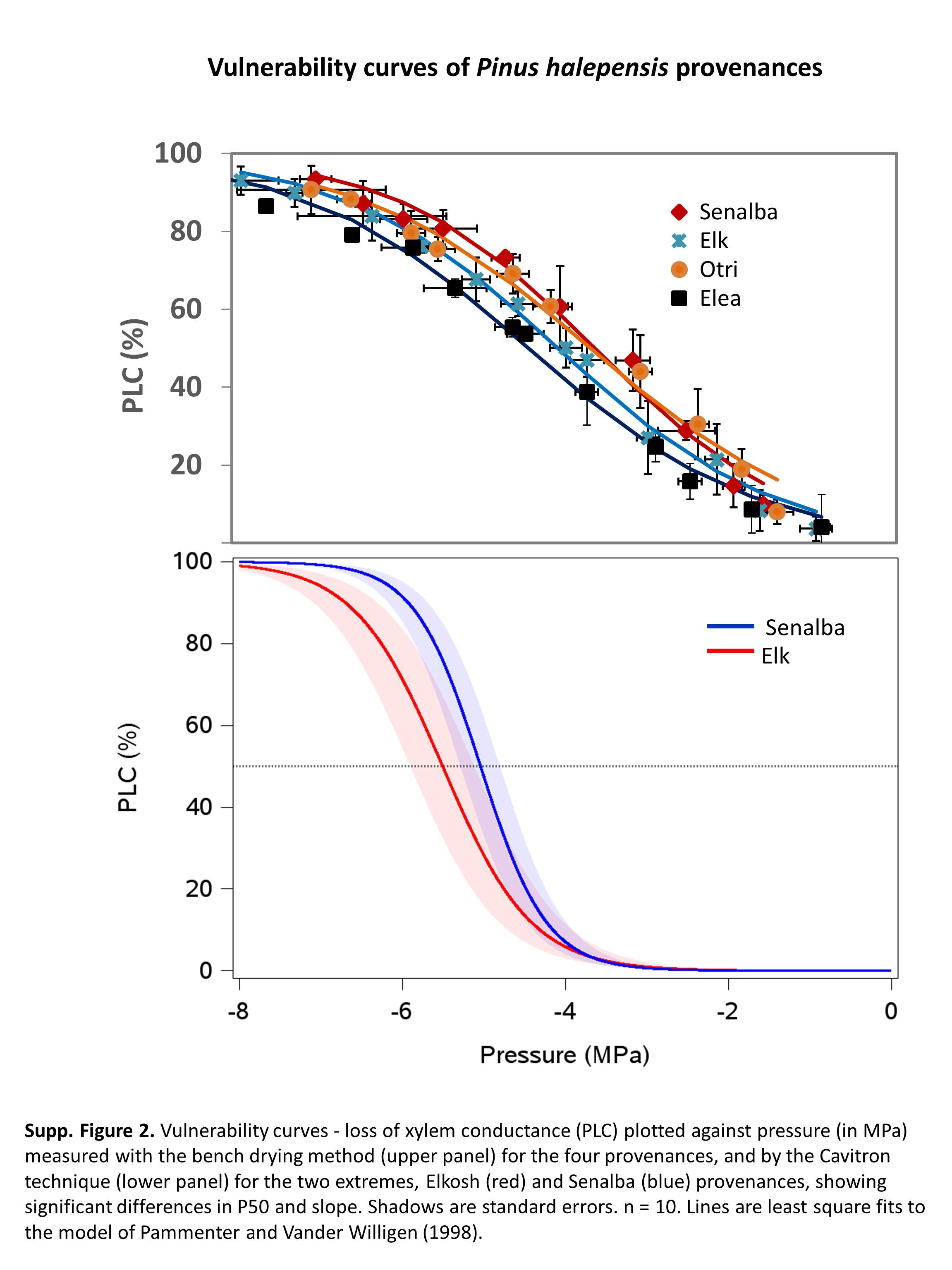

Supplement: Supplementary file 2 [file Image_2.JPEG]

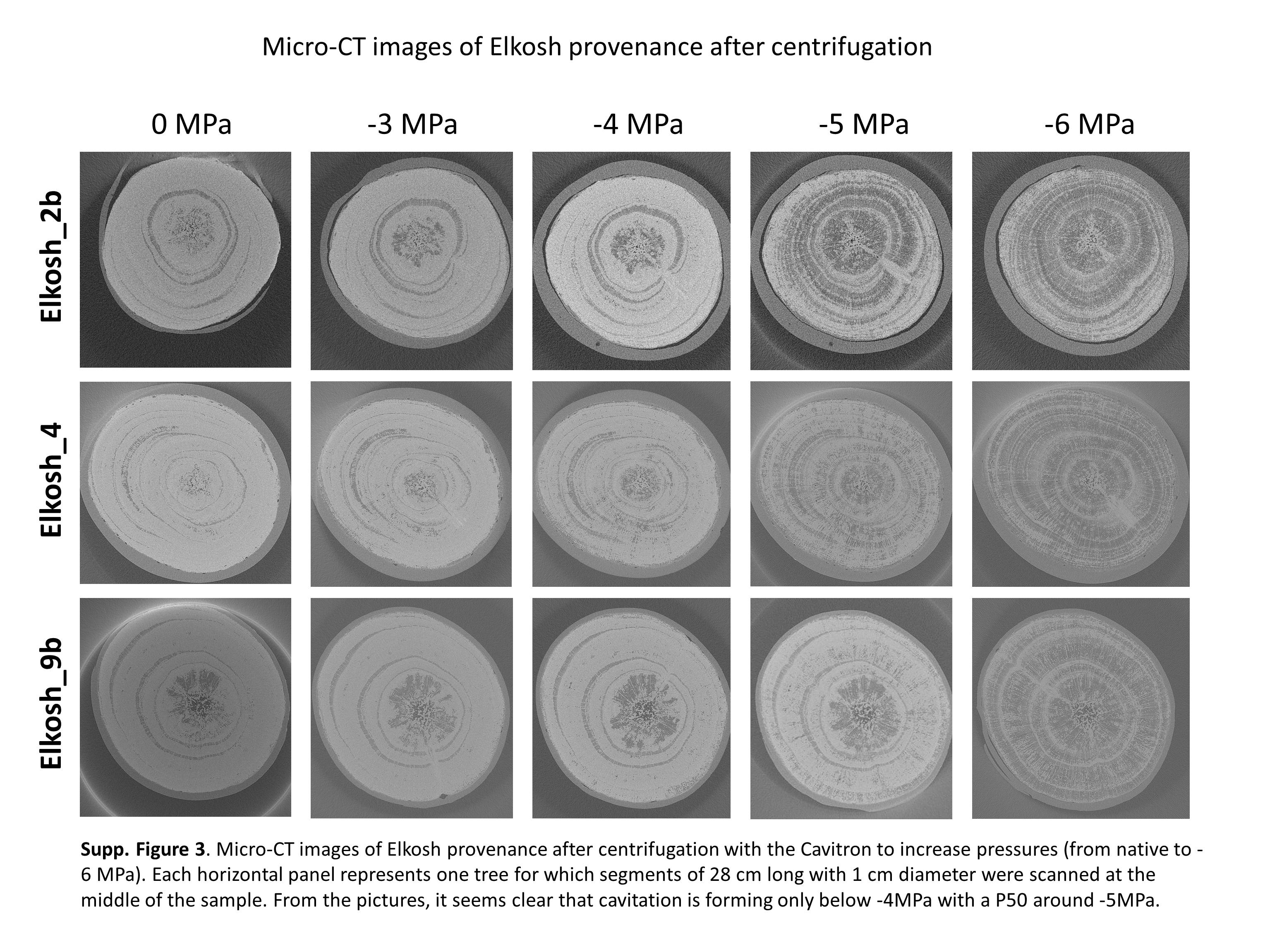

Supplement: Supplementary file 3 [file Image_3.JPEG]
